# Corticosteroid Use and Risk of Adverse Events in Metastatic Hormone‐Sensitive Prostate Cancer

**DOI:** 10.1002/pros.70143

**Published:** 2026-02-10

**Authors:** Umang Swami, Qiujun Shao, Tamuno Alfred, Maelys Touya, Frank Cao, Pinal Kamdar, Jasmina Ivanova, Johanna Celli, David Nimke

**Affiliations:** ^1^ Division of Medical Oncology, Department of Internal Medicine, Huntsman Cancer Institute University of Utah Salt Lake City Utah USA; ^2^ Astellas Pharma Global Development Inc. Northbrook Illinois USA; ^3^ Pfizer Inc. New York New York USA; ^4^ ADVI Health Washington DC USA

**Keywords:** adverse events, corticosteroids, metastatic hormone‐sensitive prostate cancer, treatment patterns

## Abstract

**Background:**

Among the approved therapies for metastatic hormone‐sensitive prostate cancer (mHSPC), abiraterone and docetaxel are administered concomitantly with corticosteroids. This study evaluated the association between corticosteroid use and risk of adverse events among patients with mHSPC.

**Methods:**

We conducted an observational cohort study among patients ≥ 65 years of age who were enrolled in Medicare parts A, B, or D, and initiated treatment for mHSPC. The primary exposure was corticosteroids between June 1, 2017–December 31, 2023. Primary outcomes were nine composite adverse event outcomes. Exploratory outcomes were all‐cause death, all‐cause hospitalization, and adverse event‐related hospitalization. Conventional Cox models were used to model corticosteroid exposure as a binary time‐varying covariate, and weighted cumulative exposure models were used to account for varying doses, durations, and timing of corticosteroid exposure.

**Results:**

Of 24,857 patients, 12,839 (52%) received at least one dose of corticosteroids during the follow‐up period. Compared with patients who were not exposed to at least one ≥ 5 mg prednisone‐equivalent dose of corticosteroids, patients exposed to corticosteroids during follow‐up were at significantly higher risk of all adverse event types, except ophthalmic events, and had a 34% higher risk of all‐cause death (adjusted hazard ratio: 1.34; 95% confidence interval: 1.27–1.42). Risks increased with prolonged corticosteroid exposure and with higher daily doses.

**Conclusions:**

Our findings suggest that patients exposed to corticosteroids are at increased risk of adverse events, hospitalization, and death. As not all mHSPC treatments require concomitant use of corticosteroids, these findings may help to inform treatment decision‐making.

## 1 Introduction

Treatment for patients with metastatic hormone‐sensitive prostate cancer (mHSPC) has evolved from androgen‐deprivation therapy (ADT) alone to include a broad range of treatment options. One recommended treatment option is combination therapy, which consists of ADT with androgen receptor pathway inhibitors (ARPIs; e.g., abiraterone, apalutamide, darolutamide, enzalutamide), and may also include docetaxel [1, 2]. Per prescribing information, abiraterone and docetaxel are required to be administered concomitantly with corticosteroids [3, 4, 5]. By reducing inflammation and compensating for reductions in serum cortisol, this co‐administration can help to reduce both cancer‐related symptoms (e.g., pain, fatigue) and treatment‐related adverse events (AEs; e.g., low potassium levels, edema, hypersensitivity) [6, 7, 8].

Despite these benefits, previous studies have indicated that corticosteroid exposure, especially long‐term exposure, is also associated with AEs such as glaucoma, diabetes, and hypertension [8, 9, 10, 11]. In a 2020 real‐world study of patients with castration‐resistant prostate cancer (CRPC), patients who received corticosteroids had a significantly higher risk of developing infections, peptic ulcers, acute cardiovascular events, endocrine disorders, or fractures than those not receiving corticosteroids [12].

Although an association between corticosteroid use and AEs has previously been demonstrated in patients with CRPC, this association has not yet been evaluated among patients with mHSPC. In addition, patterns of corticosteroid use in this population have not been well described. The primary objective of this study was to assess the association between corticosteroid exposure and risk of developing AEs among patients with mHSPC in the United States (US) Medicare population. Corticosteroid treatment patterns were evaluated as a secondary objective.

## 2 Methods

This was an observational cohort study using secondary data from the Centers for Medicare and Medicaid Services (CMS). Data were collected from beneficiaries enrolled in Medicare parts A, B, and D. Medicare is a US national program that provides access to health insurance for Americans 65 years of age or older, certain disabled patients, and patients with end‐stage renal disease. In addition to enrollment and beneficiary demographics, the data contains information about diagnostic and procedure codes, medications dispensed, dates of service, place of service, type of provider, and costs paid by Medicare. Part A and B data have a lag of 5 to 6 months and are updated on a quarterly basis. Part D data are updated on an annual basis.

Eligible patients were ≥ 65 years of age (reflective of Medicare eligibility) and initiated treatment for mHSPC (i.e., ADT, enzalutamide, apalutamide, abiraterone, docetaxel, darolutamide, or nonsteroidal antiandrogens [i.e., bicalutamide, flutamide, nilutamide]) after June 1, 2017 (the date on which data on the efficacy of abiraterone for mHSPC were made publicly available). Full eligibility criteria are presented in Supporting Information S1: Table 1.

Index date was defined as the date of systemic treatment initiation for mHSPC. Baseline patient and clinical characteristics (e.g., demographics, index year, site of metastatic disease, comorbidities, prior corticosteroid use, and healthcare resource utilization) were measured for 1 year prior to index date, except prior corticosteroid use, which was measured using all available lookback.

Corticosteroid exposure was assessed during follow‐up. Observed daily doses were standardized to prednisone‐equivalent (PE) doses to account for the fact that corticosteroids differ in glucocorticoid activity (e.g., 20 mg hydrocortisone is equivalent to 5 mg prednisone) [9]. Prescription claims for oral corticosteroids were transformed into a log of daily doses based on prescription fill date, days of supply, dose, and quantity dispensed. Consecutive prescriptions of the same drug and dose with a gap of ≤ 30 days were bridged. Prescriptions with overlapping dates were appended if they had the same drug and dose, but prescriptions of a different drug and/or dose were considered prescription replacements. For the purposes of this study, only oral corticosteroids were evaluated [12].

The primary outcomes were nine composite outcomes that were selected based on their prevalence and clinical importance among older patients: cardiovascular, dermatological, endocrine and metabolic, fluid and electrolyte disturbance, gastrointestinal, hematologic, musculoskeletal, ophthalmic, and infection events [9, 11, 12]. These outcomes are defined in greater detail in Supporting Information S1: Table 2.

Exploratory outcomes were all‐cause death, all‐cause hospitalization, and AE‐related hospitalization. To investigate how the effect of corticosteroid exposure on AE risk is impacted by age, we also explored associations by age group (≥ 65 to < 75 years, ≥ 75 years).

Patients were censored when they switched to a different treatment, were diagnosed with metastatic CRPC (mCRPC), died (except in the analysis of all‐cause death), reached the end of enrollment, or reached the end of data availability (December 31, 2023).

### 2.1 Statistical Analyses

Descriptive statistics were used to summarize baseline patient characteristics and corticosteroid utilization. Multivariable Cox proportional hazards models were used to assess the association between corticosteroid exposure and risk of AEs (primary analysis), death, and hospitalizations (exploratory analysis). Two approaches were used for these analyses to evaluate whether a relationship between corticosteroid use and AEs exists, and how this relationship may be impacted by corticosteroid exposure duration and dose.

In the first method, conventional Cox models were used to model corticosteroid exposure as a binary time‐varying covariate (exposure to at least 1 daily dose ≥ 5 mg PE; exposed vs not (yet) exposed). Patient baseline characteristics were included as covariates.

In the second method, we used a weighted cumulative exposure (WCE) approach to account for varying doses, durations, and timing in corticosteroid exposure. This method models the cumulative effects of time‐varying exposures, weighted by recency [13]. Weight functions were constrained to smoothly decrease to zero at the end of the exposure time windows. Windows of 6 months, 1 year, and 3 years were considered to determine the time from the last corticosteroid exposure after which there would be no impact on the risk of developing the AE. For each exposure time window, 1 to 3 interior knots were assessed for the spline function for the most common AE (cardiovascular) and the best‐fitting model was identified by the lowest Akaike information criterion. The same window (1 year) and number of knots (1 knot) were then used to model all other AE outcomes and death. Hazard ratios and bootstrap 95% confidence intervals (CIs) were generated, adjusting for patient baseline characteristics for three patterns of corticosteroid use compared to non‐use in the last year: current daily use of 5 mg PE for the last 3 months, current daily use of 10 mg PE for the last 3 months, and current daily use of 5 mg PE for the last 1 year.

We used a multivariable Andersen‐Gill extension of the Cox model to evaluate the risk of all‐cause hospitalizations and AE‐related hospitalizations, as these outcomes can occur multiple times. Robust standard errors were used to generate 95% CIs. These analyses were conducted in patients not already in the hospital at index date and days spent in hospital were removed from a patient's time at‐risk.

For the exploratory analysis of effects by age group, the conventional Cox model was used with the inclusion of an interaction term between the binary time‐varying corticosteroid exposure and age group.

To evaluate whether AEs may be the results of side effects caused by docetaxel rather than corticosteroids, we also conducted a sensitivity analysis where patients who initiated treatment with docetaxel were censored on the date they started docetaxel. For this analysis, only those AEs most known to be associated with docetaxel exposure were evaluated: dermatologic, fluid and electrolyte disturbances, gastrointestinal, hematologic, and infection events [5].

All analyses were conducted using SAS software version 9.4 (SAS Institute, Cary, NC).

### 2.2 Ethics Statement

As all analyses were based on de‐identified CMS Medicare Data, this study was exempt from review by an Institutional Review Board. This study was conducted in accordance with the Declaration of Helsinki.

## 3 Results

### 3.1 Baseline Characteristics and Corticosteroid Treatment Patterns

Overall, 24,857 patients with mHSPC were included in the analysis (Table 1). The mean age for the overall cohort was 77.7 years (standard deviation [SD], 7.5 years). Patients exposed to at least one dose ≥ 5 mg PE of corticosteroids during follow‐up tended to be younger than patients who were not exposed to corticosteroids (mean age 76.8 years vs. 78.6 years, respectively). Patients exposed to corticosteroids tended to have fewer prior hospitalizations and emergency room visits, and generally fewer comorbidities (e.g., history of heart failure, diabetes, fluid and electrolyte disturbances, and anemia) in the year prior to index date than those not exposed to corticosteroids (Table 1, Supporting Information S1: Tables 3–4). The median overall follow‐up was 0.9 years (interquartile range [IQR]: 0.3, 1.9).

**Table 1:** Patient baseline characteristics by corticosteroid exposure status during follow‐up.

	Study cohort *N* = 24,857 *n* (%)	Not exposed to CS^a^ *n* = 12,179 *n* (%)	Exposed to CS^a^ *n* = 12,678 *n* (%)
Age in years at index, Mean/SD	77.7 (7.52)	78.6 (7.80)	76.8 (7.13)
Age group in years			
65–74	9538 (38)	4199 (35)	5339 (42)
75+	15,319 (62)	7980 (66)	7339 (58)
Race/ethnicity			
Hispanic (any race)	388 (2)	234 (2)	154 (1)
Non‐Hispanic Black	2381 (10)	1344 (11)	1037 (8)
Non‐Hispanic White	20,657 (83)	9904 (81)	10,753 (85)
Other/Unknown	1431 (6)	697 (6)	734 (6)
Geographic region at index			
Midwest	5964 (24)	2818 (23)	3146 (25)
Northeast	5467 (22)	2650 (22)	2817 (22)
Other/Unknown	51 (0)	24 (0)	27 (0)
South	7990 (32)	3878 (32)	4112 (32)
West	5385 (22)	2809 (23)	2576 (20)
CCI score			
Mean (SD)	7.0 (3.19)	7.0 (3.34)	7.1 (3.05)
Metastatic site			
Bone and node only	815 (3)	347 (3)	468 (4)
Bone only	9951 (40)	4575 (38)	5376 (42)
Node only	1,195 (5)	557 (5)	638 (5)
No prior metastasis	9097 (37)	4714 (39)	4383 (35)
Other (including unknown)	1800 (7)	972 (8)	828 (7)
Viscera	1999 (8)	1014 (8)	985 (8)
COVID‐19 infection	1434 (6)	830 (7)	604 (5)
Adverse events			
Cardiovascular events	21,412 (86)	10,623 (87)	10,789 (85)
Dermatologic events	4705 (19)	2297 (19)	2408 (19)
Endocrine and metabolic events	21,823 (84)	10,188 (84)	10,635 (84)
Fluid and electrolyte disturbance events	6762 (27)	3731 (31)	3031 (24)
Gastrointestinal events	555 (2)	311 (3)	244 (2)
Hematologic events	10,049 (40)	5285 (43)	4764 (38)
Musculoskeletal events	4544 (18)	2220 (18)	2324 (18)
Ophthalmic events	8792 (35)	263 (35)	4529 (36)
Infection events	3491 (14)	1904 (16)	1587 (13)
All‐cause hospitalization	10,852 (44)	5813 (48)	5039 (40)
All‐cause emergency room encounters	10,328 (42)	5188 (43)	5140 (41)
Musculoskeletal‐related medications	1496 (6)	630 (5)	866 (7)
Index treatments received (between index and + 180 days)			
ADT monotherapy	4254 (17)	2696 (22)	1558 (12)
ADT + abiraterone	1679 (7)	102 (1)	1577 (12)
ADT + apalutamide	240 (1)	147 (1)	93 (1)
ADT + enzalutamide	645 (3)	409 (3)	236 (2)
ADT + docetaxel + abiraterone	105 (0)	0	> 90 (0)
ADT + docetaxel + darolutamide	39 (0)	> 11 (0)	> 20 (0)
ADT + NSAA (bicalutamide, flutamide, or nilutamide	6347 (26)	3281 (27)	3066 (24)
Other^b^	11,548 (47)	5521 (45)	6027 (48)
Average daily dose of CS (over all available lookback prior to index)^c^			
No exposure	12,031 (48)	6816 (56)	5215 (41)
≤ 0.075 mg^d^	4233 (17)	1978 (16)	2255 (18)
> 0.075 to ≤ 0.221 mg^d^	4232 (17)	1883 (15)	2349 (19)
> 0.221 mg^d^	4361 (18)	1502 (12)	2859 (23)

*Note:* Unless otherwise stated, characteristics were assessed in the 1 year prior to index.

Abbreviations: ADT, androgen‐deprivation therapy; ARPI, androgen receptor pathway inhibitor; CCI, Charlson Comorbidity Index; CS, corticosteroids; IQR, interquartile range; NSAA, non‐steroidal antiandrogen; SD, standard deviation.

^a^ Exposed to CS defined as receipt of at least one dose of ≥ 5 mg prednisone‐equivalent dose during the follow‐up period.

^b^ Other treatments included combination therapy (ADT + darolutamide, ADT + docetaxel, ARPI + NSAA, docetaxel + ARPI, docetaxel + NSAA or docetaxel + ARPI + NSAA) and monotherapy (ARPI, abiraterone, enzalutamide, apalutamide, NSAA, docetaxel).

^c^ 0.075 and 0.221 mg were the 33rd and 66th percentiles of all non‐zero doses.

^d^ Prednisone‐equivalent dose.

Corticosteroid use throughout the follow‐up period is illustrated in Table 2. A total of 12,839 patients (52%) received at least one dose of corticosteroids during the follow‐up period. Of these, nearly all (99%) received at least one dose ≥ 5 mg PE; 10,205 (79%) received at least one dose ≥ 10 mg PE. The most used corticosteroid was prednisone, which was used by 80% of patients who received corticosteroids (41% overall), followed by dexamethasone (25% of patients who received corticosteroids; 13% overall). Among patients who received at least one dose ≥ 5 mg PE, the median longest continuous corticosteroid treatment duration was 41 days (IQR, 10, 199).

**Table 2:** Corticosteroid utilization during follow‐up period.

Corticosteroid utilization	Overall *N* = 24,857
Any dose	12,839 (52)
At least one dose ≥ 5 mg^a^, *n* (%)	12,678 (51)
At least one dose ≥ 10 mg^a^, *n* (%)	10,205 (41)
Average daily dose (mg)^a^,	
Median (IQR)	0.1 (0.0, 3.1)
Drug (any dose)^b^, *n* (%)	
Hydrocortisone, *n* (%)	95 (0.4)
Prednisone, *n* (%)	10,283 (41)
Methylprednisolone, *n* (%)	1874 (8)
Dexamethasone, *n* (%)	3176 (13)
Fludrocortisone, *n* (%)	180 (1)
Proportion of days with dose ≥ 5 mg^a^, median (IQR)^c^	0.22 (0.03, 0.72)
Proportion of days with dose ≥ 10 mg^a^, median (IQR)^c^	0.05 (0.01, 0.35)
Maximum days of continuous duration with dose ≥ 5 mg^a^, median (IQR)^c^	41 (10, 199)
Maximum days of continuous duration with dose ≥ 10 mg^a^, median (IQR)^c^	14 (3, 78)

IQR, interquartile range.

^a^ Prednisone‐equivalent dose.

^b^ Groups are not mutually exclusive.

^c^ Among patients who received at least one dose ≥ 5 mg.

### 3.2 Association Between Corticosteroid Exposure and Adverse Events

**Figure 1:**
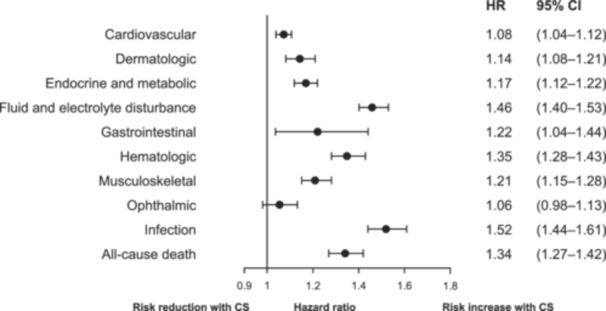
Adjusted associations between corticosteroid exposure and adverse events during follow‐up: binary time‐varying conventional Cox model. CS exposure (at least 5 mg prednisone equivalent) vs not (yet) exposed. CI, confidence interval; CS, corticosteroid; HR, hazard ratio.

Using the binary time‐varying conventional Cox model, patients exposed to at least one dose ≥ 5 mg PE of corticosteroids were at significantly higher risk of all categories of AEs compared to those not exposed to at least one dose ≥ 5 mg PE of corticosteroids, except ophthalmic AEs (Figure 1). The greatest increase in risk was observed for infections, fluid and electrolyte disturbances, and hematologic AEs (Figure 1).

**Figure 2:**
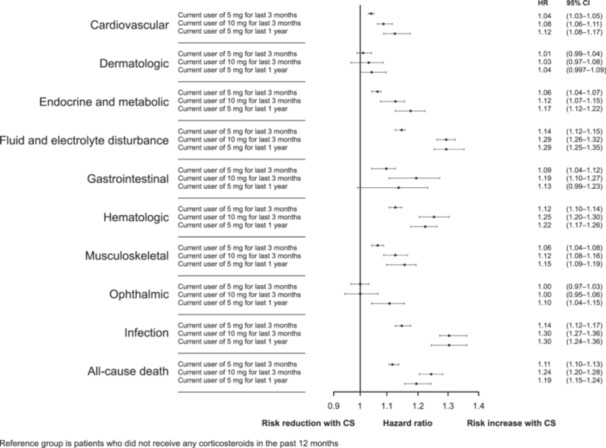
Adjusted associations between corticosteroid exposure and adverse events during follow‐up: weighted cumulative exposure model. Reference: non user in the last 1 year. All doses are prednisone equivalent. CI, confidence interval; CS, corticosteroid; HR, hazard ratio.

The results of the WCE models were consistent with the conventional approach and additionally indicated that, compared to non‐use in the last year, a daily dose of 10 mg PE corticosteroids for the last 3 months was associated with a higher risk of all types of AEs, except dermatologic and ophthalmic events. Moreover, these risks were numerically higher than the risks associated with a daily dose of 5 mg PE for the last 3 months. Furthermore, compared to non‐use in the last year, a daily dose of 5 mg PE over the last year was associated with a higher risk of all AE types except dermatologic and gastrointestinal events (Figure 2). These risks were also numerically similar or greater than those associated with a daily dose of 10 mg PE in the last 3 months for all AE types, except gastrointestinal and hematologic.

As an illustrative example of the consistency between the conventional Cox model and the WCE model, corticosteroid exposure was associated with an increased risk of musculoskeletal events in both models. At a 5 mg PE dose, compared with patients unexposed to corticosteroids, corticosteroid exposure increased the risk of musculoskeletal events by 8% in the time‐varying conventional Cox model. In the WCE models, compared with patients who were not exposed to corticosteroids in the past 12 months, treatment with 5 mg PE over the past 3 months increased the risk of musculoskeletal AEs by 6%; treatment with 10 mg PE over the past 3 months increased the risk of events 12%; and treatment with 5 mg PE for the past 12 months increased the risk of events by 15%.

In the exploratory analyses, no apparent interaction between corticosteroid exposure and age was identified, as the increased risk of AEs among patients exposed to corticosteroids remained consistent across age groups (Supporting Information S1: Figure 1). This increased risk was also present after censoring patients who initiated first‐line treatment with docetaxel (Supporting Information S1: Figure 2).

### 3.3 All‐Cause and Adverse Event‐Related Hospitalization

Using the binary time‐varying conventional Cox model, patients who were exposed to at least one dose of ≥ 5 mg PE corticosteroids were at significantly higher risk for both all‐cause hospitalization (adjusted hazard ratio [aHR], 1.35; 95% CI, 1.29–1.41) and AE‐related hospitalization (aHR, 1.50; 95% CI, 1.40–1.61) compared to patients who were not exposed to at least one dose of ≥ 5 mg PE corticosteroids. No apparent interaction between age and corticosteroid exposure was identified (Supporting Information S1: Figure 3).

### 3.4 All‐Cause Death

Using the binary time‐varying conventional Cox model, patients exposed to at least one dose of ≥ 5 mg PE corticosteroids had a 34% higher risk of all‐cause death compared to patients who were not exposed to at least one dose of ≥ 5 mg PE corticosteroids (aHR, 1.34; 95% CI, 1.27–1.42) (Figure 1). This finding was also present using the WCE model, which found that, compared with non‐use over the last year, daily corticosteroid doses of 5 and 10 mg PE for the last 3 months and daily doses of 5 mg PE for the last 1 year were associated with an increased risk of all‐cause death (Figure 2). Using the conventional Cox model, exposure to at least one dose of 5 mg PE corticosteroids was associated with a more than 30% increase in the risk of all‐cause death in both age groups (Supporting Information S1: Figure 1).

## 4 Discussion

In this analysis of patients in the CMS Medicare database, patients with mHSPC who were exposed to ≥ 5 mg PE corticosteroids were at significantly higher risk of developing cardiovascular, dermatologic, endocrine and metabolic, fluid and electrolyte disturbance, gastrointestinal, hematologic, musculoskeletal, and infection AEs than those not exposed to corticosteroids. Moreover, patients exposed to corticosteroids were at increased risk of hospitalization and death. These associations were present among both patients ≥ 65 to < 75 years and patients ≥ 75 years of age and remained even after censoring patients who initiated treatment with docetaxel.

This study is the first to evaluate how corticosteroid exposure impacts AE risk among patients with mHSPC. Overall, this study highlights the increased risk of AEs associated with corticosteroid exposure among patients with mHSPC, particularly among patients with long‐term exposure. Our findings are consistent with studies conducted among patients with mCRPC, which also found an increased risk of AEs among patients who received corticosteroids [8, 12, 14]. Overall, patients in this study who were exposed to at least one dose of ≥ 5 mg PE corticosteroids during the follow‐up period tended to be younger, and had generally experienced fewer comorbidities and hospitalizations at baseline compared to patients who were not exposed to corticosteroids. These findings may suggest that physicians may be more cautious about prescribing corticosteroids to patients who are older and sicker, and who may respond poorly to corticosteroid exposure. As not all treatments approved for mHSPC require concomitant use of corticosteroids, avoiding unnecessary toxicity and AEs due to long‐term corticosteroid exposure may help to inform treatment decision‐making.

The use of WCE models revealed that the risk of AEs and death increased with higher daily doses of corticosteroids and when low daily doses of corticosteroids were taken for longer periods of time. Although 5 mg PE corticosteroids may be perceived as a relatively low dose of corticosteroids, our findings suggest that chronic exposure at this level is associated with increased risks for patients, consistent with findings from previous studies of long‐term corticosteroid exposure [9, 10]. Indeed, in our study, the risk of AEs (except gastrointestinal and hematologic events) was similar or greater after a longer exposure to a lower dose of corticosteroids (i.e., 5 mg PE for the last 1 year) than after shorter exposure to a higher dose (i.e., 10 mg PE for the last 3 months). This is notable, as among patients in this study who received at least one dose ≥ 5 mg PE, the median longest continuous corticosteroid treatment duration was 41 days and the 75th percentile was 199 days. Many patients treated with corticosteroids continued to be exposed for long periods of time, and this practice was associated with higher rates of adverse outcomes.

Strengths of this study include our large, representative population of patients ≥ 65 years of age who have mHSPC; our use of new modeling techniques that can account for varying doses, durations, and timing of corticosteroid exposure; and the assessment of multiple AEs as well as hospitalizations and death.

This study has limitations. Due to our use of a secondary health administrative database, the case definitions of mHSPC and baseline characteristics used in our analysis rely on administrative codes, which were not validated with a gold standard source such as chart review. As a result, it is possible that some conditions may have been misclassified. Corticosteroid exposure was based on pharmacy claims data, which may overestimate the amount of corticosteroids actually taken by the patient. Finally, although our analyses were adjusted for baseline patient and clinical variables, there is a risk of residual confounding, as some potential confounders may not be captured in our dataset. Specifically, corticosteroid exposure that occurred prior to age 65 (i.e., age of Medicare eligibility) would not have been captured in our dataset. We attempted to mitigate this issue by including history of medical conditions that are commonly treated using corticosteroids as variables in our analysis.

## 5 Conclusion

This study suggests that, among a cohort of patients receiving systemic therapy for mHSPC, corticosteroid exposure was associated with increased risk of cardiovascular, dermatologic, endocrine and metabolic, fluid and electrolyte disturbance, gastrointestinal, hematologic, musculoskeletal, and infection AEs, as well as hospitalization and death. Notably, chronic use of corticosteroids further increased the risk of AEs, even at relatively low doses. As treatment duration for mHSPC can be relatively long, the increased risk of AEs associated with long‐term corticosteroid exposure should be carefully considered when selecting a treatment pathway.

## Author Contributions

U.S. made substantial contributions to the study design and was involved with interpretation of the study data. Q.S. made substantial contributions to the study design, and was involved with the acquisition, analysis, and interpretation of the study data. T.A. made substantial contributions to the study design, and was involved with the acquisition, analysis, and interpretation of the study data. M.T. made substantial contributions to the study design, and was involved with the acquisition, analysis, and interpretation of the study data. F.C. was involved with the acquisition, analysis and interpretation of the study data. P.K. made substantial contributions to the study design, and was involved with the acquisition, analysis, and interpretation of the study data. J.I. made substantial contributions to the study design, and was involved with the acquisition, analysis, and interpretation of the study data. J.C. was involved with the acquisition and analysis of the study data. D.N. made substantial contributions to the study design, and was involved with the acquisition, analysis and interpretation of the study data.

## Ethics Statement

As all analyses were based on de‐identified CMS Medicare Data, this study was exempt from review by an Institutional Review Board. This study was conducted in accordance with the Declaration of Helsinki.

## Conflicts of Interest

U.S. reports consulting or advisory roles with Adaptimmune, Astellas Pharma, AstraZeneca, Exelixis, Flatiron Health, Kairos, Gilead Sciences, Imvax, Janssen Scientific Affairs, Pfizer, Sanofi, and Seagen; received research funding paid to institution from Exelixis, Janssen, Lava Therapeutics, Loxo/Lilly, Merck, Oric Pharmaceuticals, Seagen, and Seattle Genetics/Astellas Pharma. Q.S. reports employment in the past 2 years with Astellas Pharma and Novartis; holds stock or other ownership with Novartis. T.A. reports employment in the past 2 years with Astellas Pharma and Pfizer Inc; holds stock or other ownership with Pfizer Inc; received research funding from Astellas Pharma and Pfizer Inc. M.T. reports employment in the past 2 years with Astellas Pharma. F.C. reports employment in the past 2 years with Astellas Pharma; received research funding from Astellas Pharma. P.K. reports employment in the past 2 years with Astellas Pharma. J.I. reports employment in the past 2 years with Pfizer Inc; holds stock or other ownership with Pfizer Inc. J.C. reports employment in the past 2 years with ADVI Health. D.N. reports employment in the past 2 years with Astellas Pharma.

## Supporting information


**Supplementary Table 1:** Study cohort selection. **Supplementary Table 2:** Adverse event outcome definitions. **Supplementary Table 3:** Prior adverse events at baseline (1 year prior to index). **Supplementary Table 4:** Charlson Comorbidity Index score and conditions at baseline (1 year prior to index).

## Data Availability

Details for how researchers may request access to anonymized participant level data, trial level data and protocols from Astellas sponsored clinical trials can be found at https://www.clinicaltrials.astellas.com/transparency/.
