# Supplementary material for: Corticosteroid Use and Risk of Adverse Events in Metastatic Hormone‐Sensitive Prostate Cancer
Source: Prostate. 2026 Feb 10;86(7):763–70. doi: 10.1002/pros.70143 (PMC13077259; doi:10.1002/pros.70143)
Supplement: Supplementary file 1 — Supplementary Table 1: Study cohort selection. Supplementary Table 2: Adverse event outcome definitions. Supplementary Table 3: Prior adverse events at baseline (1 year prior to index). Supplementary Table 4: Charlson Comorbidity Index score and conditions at baseline (1 year prior to index). [file PROS-86-763-s001.docx]

**Supplementary Materials**

**Supplementary Table 1. Study cohort selection**

| **Inclusion/Exclusion Criteria** | **Patient Count** |
| --- | --- |
| 1. Patient has ≥ 1 claim for ADT, enzalutamide, apalutamide, abiraterone, docetaxel, darolutamide, NSAA (bicalutamide, flutamide, or nilutamide) | 2 301 176 |
| 2. Treatment was for patient with ≥ 1 claim with a diagnosis code for secondary metastasis | 610 458 |
| 3. Treatment date is within 30 days prior to or 90 days after the first claim for metastatic disease | 307 960 |
| 4. Patient has index date after Jun 1, 2017 | 153 625 |
| 5. Patient is male | 107 963 |
| 6. Patient is ≥ 65 years old as of the index date | 102 112 |
| 7. Patient has ≥ 1 claim with a diagnosis code in any position for prostate cancer prior to or on the index date | 80 149 |
| 8. Patient has continuous enrollment in Medicare Parts A, B, and D for ≥ 1 year before the index date | 53 110 |
| 9. Patient has continuous enrollment in Medicare Parts A, B, and D for ≥ 3 months after the index date | 52 535 |
| 10. Patient has no evidence of ARPI or surgical castration or docetaxel before index date, or other systemic prostate cancer treatment including PARP inhibitor, immunotherapy, chemotherapy, radium-223, Pluvicto, cabazitaxel, or castration resistance diagnosis at any time prior to the index date or within 30 days after the index date | 29 496 |
| 11. Patient has no evidence of index treatment or ADT during 365 days prior to the index date | 26 383 |
| 12. Patient has no claim with a code indicating participation in a clinical trial prior to index date | **24 857** |

ADT, androgen-deprivation therapy; ARPI, androgen receptor pathway inhibitor; NSAA, nonsteroidal antiandrogen therapies; PARP, poly (ADP-ribose) polymerase.

**Supplementary Table 2. Adverse event outcome definitions**

| **Adverse Event Type** | **Definition^a^** |
| --- | --- |
| Cardiovascular | - Diagnosis of hypertension without baseline history - Diagnosis of other cardiovascular events (i.e., ischemic heart disease, myocardial infarction, cerebrovascular disease, heart failure, angina/revascularization) |
| Dermatological | - Any dermatologic events (i.e., acne, dermatitis, skin atrophy, erythema, impaired wound healing, rash, striae) |
| Endocrine and metabolic | - Diagnosis of diabetes without baseline history - Diagnosis of adrenal disorders without baseline history - Diagnosis of dyslipidemia without baseline history - Diagnosis of other endocrine and metabolic events (hyperglycemia, hypoglycemia) - Diabetes treatment intensification   - Diabetes medication (at drug class level) prescription in patients without history of that medication - Cholesterol treatment or treatment intensification   - Statin prescription in patients without baseline history   - Other lipid lowering medication prescription in patients without baseline history   - Change in statin prescribing suggesting intensification based on statin treatment guidelines |
| Fluid and electrolyte disturbance | - Any fluid or electrolyte disturbance events (i.e., hypokalemia or other) |
| Gastrointestinal | - Any gastrointestinal events (i.e., peptic ulcers and related complications) |
| Hematologic | - Diagnosis of anemia without baseline history - Diagnosis of neutropenia |
| Musculoskeletal | - Diagnosis of osteoporosis without baseline history - Diagnosis of other musculoskeletal events (fractures, osteopenia, myopathy) |
| Ophthalmic | - Diagnosis of cataract without baseline history - Diagnosis of glaucoma without baseline history |
| Infection | - Any infection events (pneumonia, septicemia, bacteremia) |

^a^All definitions refer to the first occurrence of an event on or after the index date. Diagnostic codes are available from authors upon request.

**Supplementary Table 3. Prior adverse events at baseline (1 year prior to index)**

|  | **Study Cohort N = 24 857**  **n (%)** | **Not Exposed to CS in Follow-up* n = 12 179** | **Exposed to CS in Follow-up* n = 12 678** |
| --- | --- | --- | --- |
| **Cardiovascular events** | | | |
| Total | 21 412 (86) | 10 623 (87) | 10 789 (85) |
| Angina/Revascularization | 5 077 (20) | 2 535 (21) | 2 542 (20) |
| Cerebrovascular disease | 4 595 (18) | 2 426 (20) | 2 169 (17) |
| Heart failure | 4 368 (18) | 2 388 (20) | 1 980 (16) |
| Hypertension | 20 297 (82) | 10 093 (83) | 10 204 (80) |
| Ischemic heart disease | 9 709 (39) | 4 886 (40) | 4 823 (38) |
| Myocardial infarction | 1 090 (4) | 611 (5) | 479 (4) |
| **Dermatological events** | | | |
| Total | 4 705 (19) | 2 297 (19) | 2 408 (19) |
| Acne | 126 (1) | 61 (1) | 65 (1) |
| Dermatitis | 3 144 (13) | 1 542 (13) | 1 602 (13) |
| Erythema | 735 (3) | 349 (3) | 386 (3) |
| Impaired wound healing | 36 (0) | 22 (0) | 14 (0) |
| Rash | 987 (4) | 482 (4) | 505 (4) |
| Skin atrophy | 807 (3) | 376 (3) | 431 (3) |
| **Endocrine and metabolic events** | | | |
| Total | 20 823 (84) | 10 188 (84) | 10 635 (84) |
| Adrenal disorders | 577 (2) | 273 (2) | 304 (2) |
| Diabetes | 8 452 (34) | 4 385 (36) | 4 067 (32) |
| Diabetes treatment | 5 381 (22) | 2 818 (23) | 2 563 (20) |
| Dyslipidemia | 18 490 (74) | 9 027 (74) | 9 463 (75) |
| Hyperglycemia | 5 248 (21) | 2 440 (20) | 2 808 (22) |
| Hypoglycemia | 322 (1) | 183 (2) | 139 (1) |
| Statin treatment | 14 053 (57) | 6 810 (56) | 7 243 (57) |
| **Fluid and electrolyte disturbance events** | | | |
| Total | 6 762 (27) | 3 731 (31) | 3 031 (24) |
| Hypokalemia | 1 952 (8) | 1 122 (9) | 830 (7) |
| Other | 6 015 (24) | 3 333 (27) | 2 682 (21) |
| **Gastrointestinal events** | | | |
| Total | 555 (2) | 311 (3) | 244 (2) |
| **Hematologic events** | | | |
| Total | 10 049 (40) | 5 285 (43) | 4 764 (38) |
| Anemia | 10 001 (40) | 5 271 (43) | 4 730 (37) |
| Neutropenia | 182 (1) | 80 (1) | 102 (1) |
| **Musculoskeletal events** | | | |
| Total | 4 544 (18) | 2 220 (18) | 2 324 (18) |
| Fractures | 3 033 (12) | 1 493 (12) | 1 540 (12) |
| Myopathy | 162 (1) | 90 (1) | 72 (1) |
| Osteopenia | 524 (2) | 222 (2) | 303 (2) |
| Osteoporosis | 1 505 (6) | 763 (6) | 742 (6) |
| **Ophthalmic events** | | | |
| Total | 8 792 (35) | 4 263 (35) | 4 529 (36) |
| Cataract | 6 777 (27) | 3 221 (26) | 3 556 (28) |
| Glaucoma | 3 926 (16) | 1 946 (16) | 1 980 (16) |
| **Infection events** | | | |
| Total | 3 491 (14) | 1 904 (16) | 1 587 (13) |
| Bacteremia | 502 (2) | 296 (2) | 206 (2) |
| Pneumonia | 2 377 (10) | 1 249 (10) | 1 128 (9) |
| Septicemia | 1 679 (7) | 957 (8) | 722 (6) |

*Exposed to CS defined as receipt of at least one dose of ≥ 5 mg prednisone-equivalent dose during the follow-up period.

CS, corticosteroid.

**Supplementary Table 4. Charlson Comorbidity Index score and conditions at baseline (1 year prior to index)**

|  | **Study Cohort N = 24 857** | **Not Exposed to CS in Follow-up* n = 12 179** | **Exposed to CS in Follow-up* n = 12 678** |
| --- | --- | --- | --- |
| **CCI Score** | | | |
| Mean (SD) | 7.0 (3.19) | 7.0 (3.34) | 7.1 (3.05) |
| **Modified Charlson Comorbidities n (%)** | | | |
| Any malignancy** | 5 943 (24) | 3 222 (26) | 2 721 (21) |
| Cerebrovascular disease | 4 392 (18) | 2 297 (19) | 2 095 (17) |
| Congestive heart failure | 4 848 (20) | 2 595 (21) | 2 253 (18) |
| Chronic pulmonary disease | 5 876 (24) | 2 673 (22) | 3 203 (25) |
| Dementia | 1 778 (7) | 1 170 (10) | 608 (5) |
| Diabetes with chronic complication | 4 279 (17) | 2 327 (19) | 1 952 (15) |
| Diabetes without chronic complication | 4 028 (16) | 1 976 (16) | 2 052 (16) |
| HIV | 95 (0) | 51 (0) | 44 (0) |
| Metastasis | 17 418 (70) | 8 064 (66) | 9 354 (74) |
| Acute myocardial infarction | 2 706 (11) | 1 379 (11) | 1 327 (10) |
| Mild liver disease | 3 392 (14) | 1 586 (13) | 1 806 (14) |
| Moderate or severe liver disease | 168 (1) | 106 (1) | 62 (0) |
| Hemiplegia or paraplegia | 616 (2) | 303 (2) | 313 (2) |
| Peptic ulcer disease | 567 (2) | 308 (3) | 259 (2) |
| Peripheral vascular disease | 7 107 (29) | 3 664 (30) | 3 443 (27) |
| Renal disease | 6 058 (24) | 3 219 (26) | 2 839 (22) |
| Rheumatologic disease | 762 (3) | 288 (2) | 474 (4) |

*Exposed to CS defined as receipt of at least one dose of ≥ 5 mg prednisone-equivalent dose during the follow-up period.

**Cancer, excluding solid tumors with documented metastases.

CCI, Charlson Comorbidity Index; CS, corticosteroid; HIV, human immunodeficiency virus; SD, standard deviation.

**Supplementary Figure 1. Adjusted associations between corticosteroid exposure and adverse events during follow-up by age group (binary time-varying conventional Cox models)**


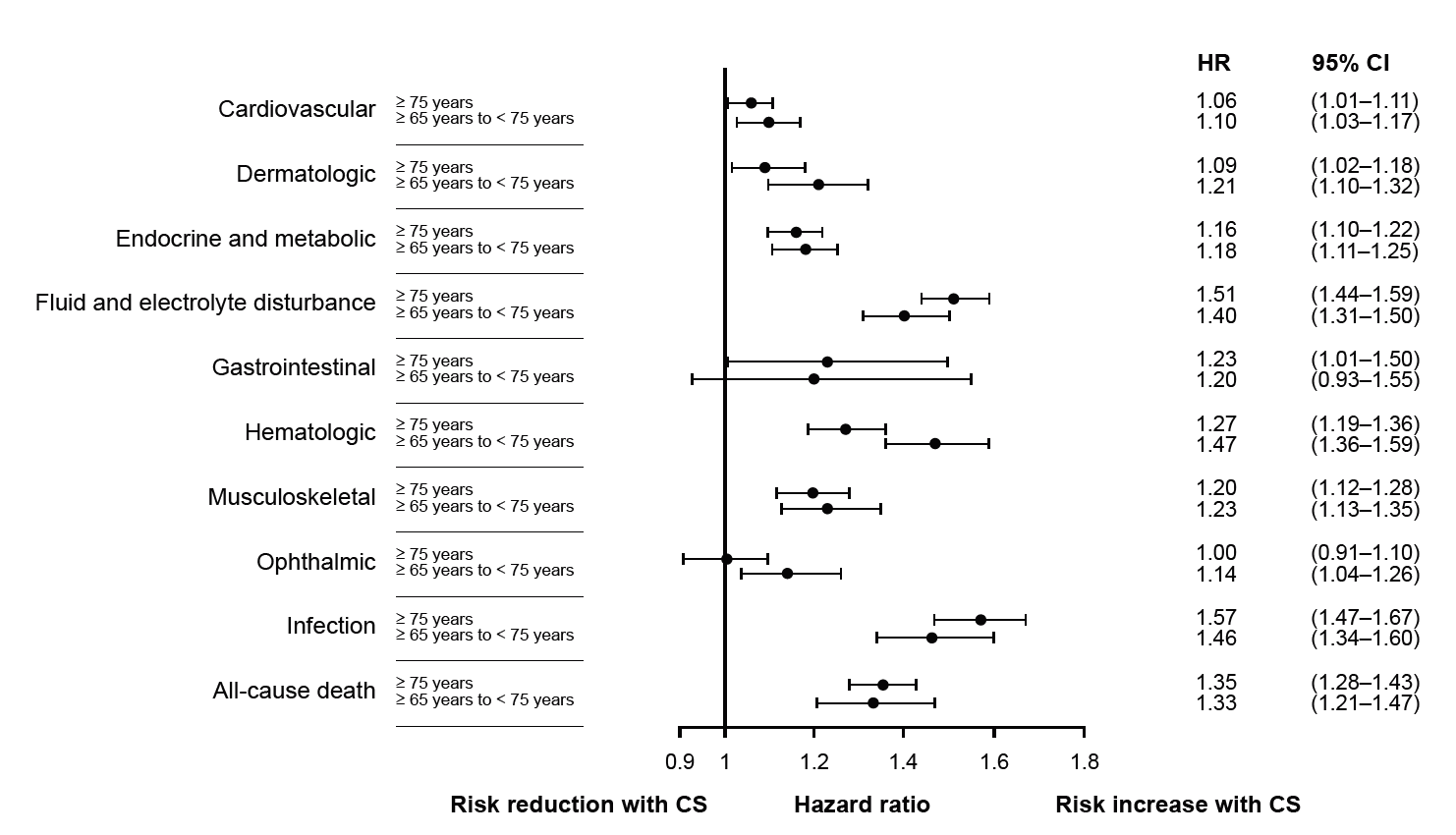


CS exposure (at least 5 mg prednisone equivalent) vs not (yet) exposed.

CI, confidence interval; CS, corticosteroid; HR, hazard ratio.

**Supplementary Figure 2. Sensitivity analysis: Adjusted associations between corticosteroid exposure and adverse events after censoring patients at time of docetaxel initiation, overall and by age group (binary time-varying conventional Cox model)**


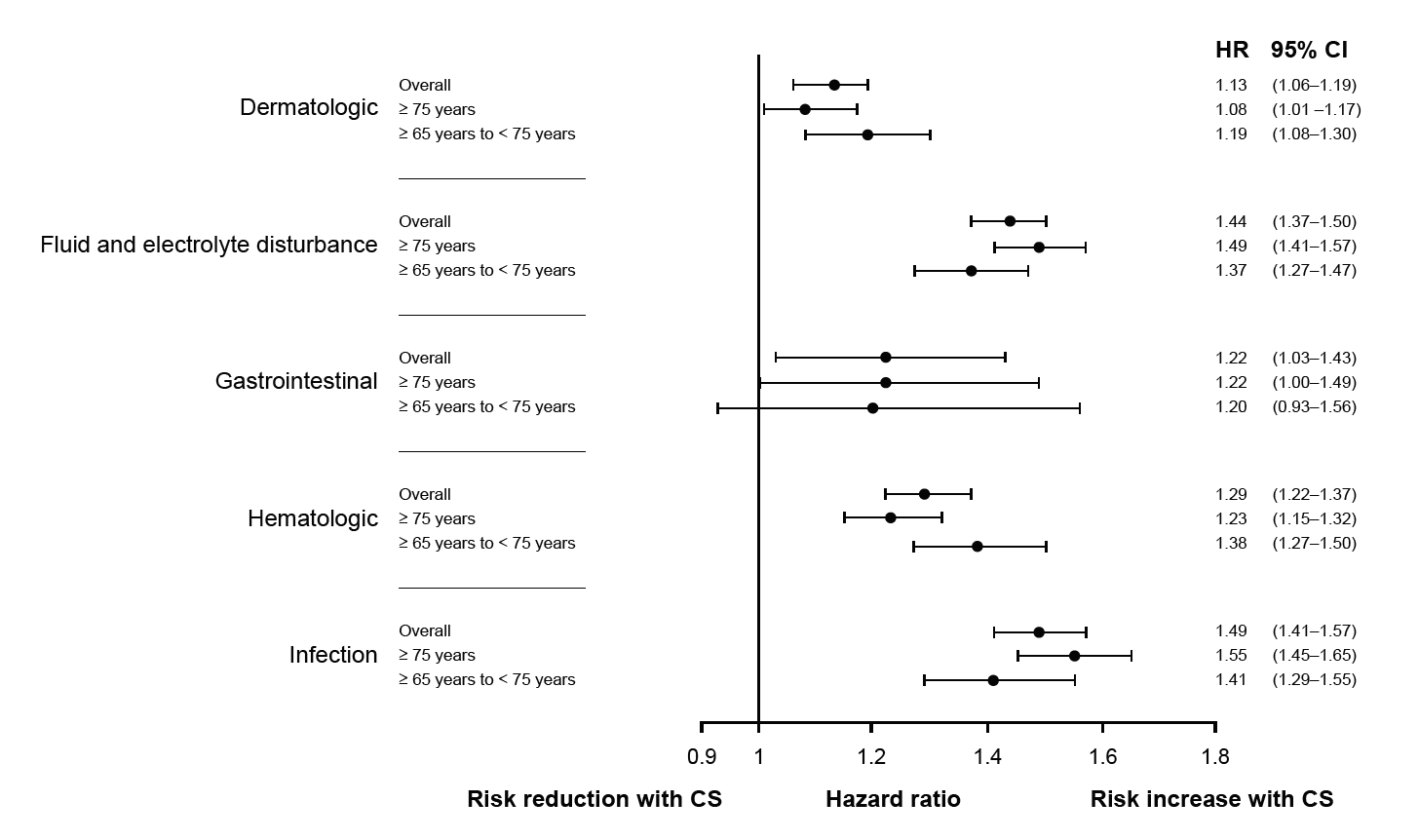


CS exposure (at least 5 mg prednisone equivalent) vs not (yet) exposed.

CI, confidence interval; CS, corticosteroid; HR, hazard ratio.

**Supplementary Figure 3. Adjusted associations between corticosteroid exposure and risk of hospitalization during follow-up, overall, and by age group (binary time-varying conventional Cox models)**


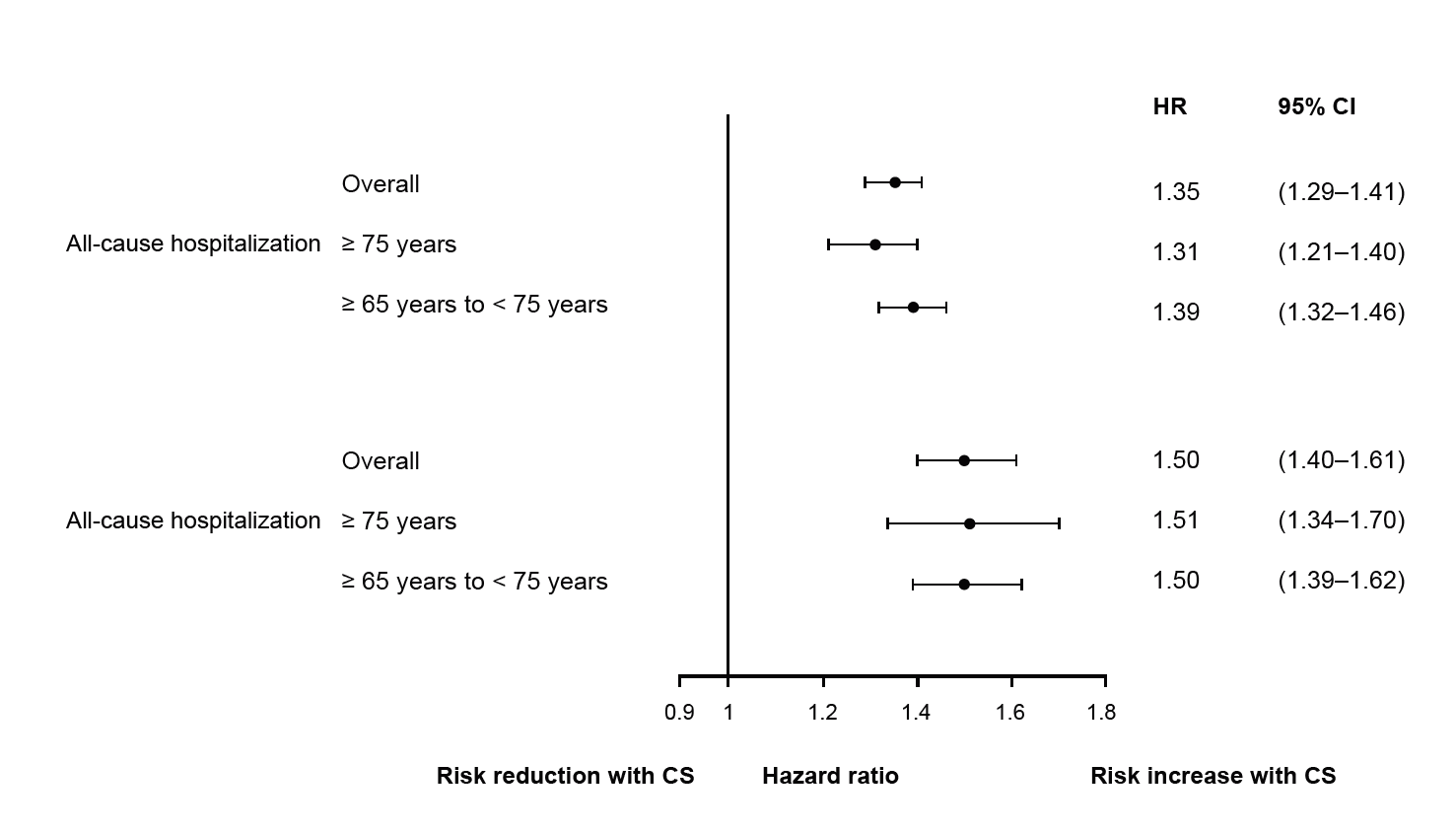


AE, adverse event; CI, confidence interval; CS, corticosteroid; HR, hazard ratio.
